# Uterus transplantation: from research, through human trials and into the future

**DOI:** 10.1093/humupd/dmad012

**Published:** 2023-06-16

**Authors:** Mats Brännström, Catherine Racowsky, Marie Carbonnel, Joseph Wu, Antonio Gargiulo, Eli Y Adashi, Jean Marc Ayoubi

**Affiliations:** Department of Obstetrics and Gynecology, Sahlgrenska Academy, University of Gothenburg, Gothenburg, Sweden; Stockholm IVF-EUGIN, Stockholm, Sweden; Department of Obstetrics, Gynecology and Reproductive Medicine, Hospital Foch, Suresnes, France; Department of Obstetrics, Gynecology and Reproductive Medicine, Hospital Foch, Suresnes, France; Department of Obstetrics, Gynecology and Reproductive Biology, Brigham and Women’s Hospital, Harvard Medical School, Boston, MA, USA; Department of Obstetrics, Gynecology and Reproductive Medicine, Hospital Foch, Suresnes, France; University Versailles, Saint-Quentin en Yvelines, France; Warren Alpert Medical School, Brown University, Providence, RI, USA; Department of Obstetrics, Gynecology and Reproductive Biology, Brigham and Women’s Hospital, Harvard Medical School, Boston, MA, USA; Department of Medical Science, Brown University, Providence, RI, USA; Department of Obstetrics, Gynecology and Reproductive Medicine, Hospital Foch, Suresnes, France; University Versailles, Saint-Quentin en Yvelines, France

**Keywords:** ethics, surgery, assisted reproduction, IVF, infertility, uterine factor infertility, human, animal models, transplantation, uterus

## Abstract

Women suffering from absolute uterine factor infertility (AUFI) had no hope of childbearing until clinical feasibility of uterus transplantation (UTx) was documented in 2014 with the birth of a healthy baby. This landmark accomplishment followed extensive foundational work with a wide range of animal species including higher primates. In the present review, we provide a summary of the animal research and describe the results of cases and clinical trials on UTx. Surgical advances for graft removal from live donors and transplantation to recipients are improving, with a recent trend away from laparotomy to robotic approaches, although challenges persist regarding optimum immunosuppressive therapies and tests for graft rejection. Because UTx does not involve transplantation of the Fallopian tubes, IVF is required as part of the UTx process. We provide a unique focus on the intersection between these two processes, with consideration of when oocyte retrieval should be performed, whether, and for whom, preimplantation genetic testing for aneuploidy should be used, whether oocytes or embryos should be frozen and when the first embryo transfer should be performed post-UTx. We also address the utility of an international society UTx (ISUTx) registry for assessing overall UTx success rates, complications, and live births. The long-term health outcomes of all parties involved—the uterus donor (if live donor), the recipient, her partner and any children born from the transplanted graft—are also reviewed. Unlike traditional solid organ transplantation procedures, UTx is not lifesaving, but is life-giving, although as with traditional types of transplantation, costs, and ethical considerations are inevitable. We discuss the likelihood that costs will decrease as efficiency and efficacy improve, and that ethical complexities for and against acceptability of the procedure sharpen the distinctions between genetic, gestational, and social parenthood. As more programs wish to offer the procedure, we suggest a scheme for setting up a UTx program as well as future directions of this rapidly evolving field. In our 2010 review, we described the future of clinical UTx based on development of the procedure in animal models. This Grand Theme Review offers a closing loop to this previous review of more than a decade ago. The clinical feasibility of UTx has now been proved. Advancements include widening the criteria for acceptance of donors and recipients, improving surgery, shortening time to pregnancy, and improving post-UTx management. Together, these improvements catalyze the transition of UTx from experimental into mainstream clinical practice. The procedure will then represent a realistic and accessible alternative to gestational surrogacy for the treatment of AUFI and should become part of the armamentarium of reproductive specialists worldwide.

## Graphical abstract

**Figure:**
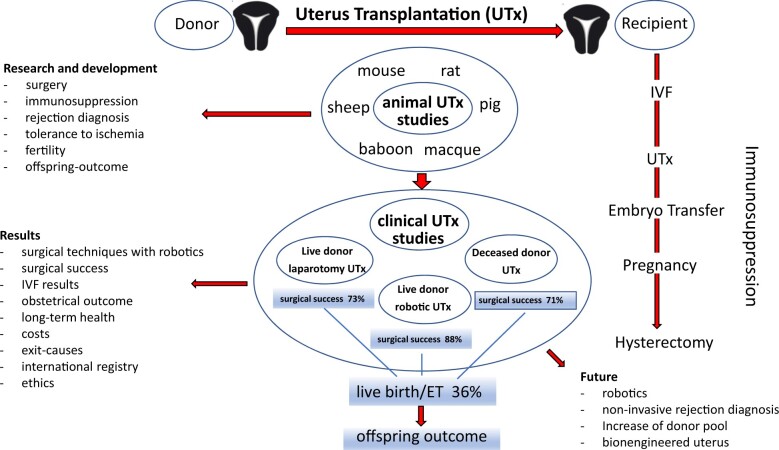
**Live birth is achievable after uterus transplantation: key features in the development and application of uterus transplantation to human. ET: embryo transfer**.

## Introduction

During the last decade, uterus transplantation (UTx) has evolved as a treatment for absolute uterine factor infertility (AUFI). The AUFI condition, affecting 1:500 women of fertile age (Sieunarine *et al.*, 2005), is caused by either uterine absence (surgical/congenital) or a uterine defect (anatomic/functional). The different causes of AUFI and their prevalence have recently been reviewed (Hur *et al.*, 2019).

**Figure 1.**
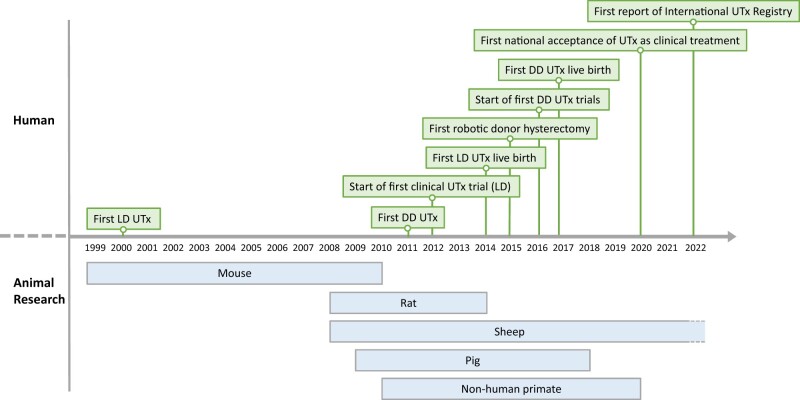
**Time-line of animal-based research and major accomplishments in human uterus transplantation**. Animal research in uterus transplantation followed a natural sequence, from rodents to large domestic species, and then to non-human primates. The specific references connected to the start of research in each animal species are: mouse (Racho El-Akouri *et al.*, 2002), rat (Wranning *et al.*, 2008a), sheep (Wranning *et al.*, 2008b), pig (Avison *et al.*, 2009), non-human primate (Enskog *et al.*, 2010). UTx, uterus transplantation; LD, live donor; DD, deceased donor.

The chronological sequences of the most significant milestones in human UTx, and the foundational animal research performed in this field, reveal that the clinical activities within human UTx have gradually evolved and were preceded by systematic animal-based research, which has continued during the experimental phase of UTx (Fig. 1). As shown, the first human UTx attempt was performed in a live donor (LD) in 2000 but this was surgically unsuccessful and a necrotic uterus was removed 3 months later (Fageeh *et al.*, 2002). The first deceased donor (DD) UTx procedure was performed in 2011, but it took multiple embryo transfer (ET) attempts and corrective surgery over several years before a birth occurred, more than 9 years later (Ozkan *et al.*, 2022). The first clinical UTx trial was undertaken in Sweden. Nine LD UTx procedures were performed from 2012 to 2013 (Brännström *et al.*, 2014), from which the landmark first live birth occurred in Sweden in 2014 (Brännström *et al.*, 2015). In 2015, minimal invasive surgery (MIS) was introduced with the first robotic-assisted LD hysterectomy, which was performed in China (Wei *et al.*, 2017). In 2016, the first clinical trials of DD UTx were started and were independently performed in the USA (Flyckt *et al.*, 2016; Testa *et al.*, 2020) and in the Czech Republic (Fronek *et al.*, 2021). The first DD UTx live birth took place in 2017 in Brazil (Ejzenberg *et al.*, 2019).

More than 80 UTx procedures have been performed in almost 20 centers in Europe, North America, Latin America, and Asia and more than 40 live births had been achieved by 2022 (Johannesson *et al.*, 2022; Brännström *et al.*, 2023). Uterus transplantation is now becoming established as a clinical treatment. It has been accepted into a national health system (Germany in 2020) (Brännström *et al.*, 2022b) and an international quality registry of UTx activities was launched by the International Uterus Transplantation Society (ISUTx) in 2020, with the first report from the registry published in 2023 (Brännström *et al.*, 2023).

As with the establishment of many clinical procedures, extensive animal research was performed to develop the safety and efficacy of the technique prior to the first clinical trials. Thus, introduction of UTx within clinical trials in the human setting has followed the structured Idea, Development, Exploration, Assessment and Long-term follow-up (IDEAL) concept (McCulloch *et al.*, 2009) and the Moore Criteria (Moore, 2000) for safe introduction of a major surgical procedure, and has also complied with the first ethical guidelines of UTx (Milliez, 2009).

In this review, we present the important underlying animal research in UTx and summarize results of published human cases with a special focus on surgical results and ART in relation to UTx. Additionally, we cover ethics, exit strategies and costs of UTx. In the final section of the review, we suggest a scheme for setting up a UTx program as well as future directions of this rapidly evolving field.

## Key animal studies

Extensive animal research paved the way for introduction of UTx in the human (Fig. 1). The findings up to 2010 have been described in detail in a review on experimental UTx (Brännström *et al.*, 2010). Here, we briefly summarize the animal studies, both pre- and post-2010, that focused on fertility after UTx, as well as the more recent work of special impact. The key-animal findings and their implications in the development toward human UTx are summarized in Table 1.

**Table 1.** Milestones reached from animal studies in the development of clinical uterus transplantation.

Milestone	Animal model	Outcomes assessed	Evidence	Reference
Proof-of-principle that UTx is feasible	Syngeneic mouse	Pregnancy post blastocyst transfer to grafted and native uteri	Pregnancy in both grafted and native uteri	Racho El-Akouri *et al.* (2002)

Implantation and miscarriage rates no different from native uterus; birthweights of second generation offspring normal	Syngeneic mouse	Live birth of second generation offspring post-blastocyst transfer	Pregnancies in eight grafts with normal growth trajectories to eight weeks and normal birthweights of second generation offspring	Racho El-Akouri *et al.* (2003a)

Graft tolerates CIT of up to 24 h; possible tissue damage is reversible	Syngeneic mouse	Growth trajectories of offspring post CIT of 24 h vs 48 h	CIT of 24 h, not 48 h, supported normal pregnancies	Racho El Akouri *et al.* (2003b)

High doses of cyclosporine decrease pregnancy rate	Mouse	Dose-response effect of cyclosporine for IS on pregnancy	25% decreased implantation rate and 2-fold increased miscarriage rate at >10 mg/kg/day cyclosporine	Groth *et al.* (2010)

Pregnancy achievable in allogeneic model post-UTx using Tac for IS	Allogeneic rat	Pregnancy post-UTx after natural mating	Pregnancy occurred but mean number of pups lower in Tac groups	Diaz-Garcia *et al.* (2010)

Allogeneic uterine grafts, with Tac IS can result in offspring with normal postnatal growth	Allogeneic rat	Effect of Tac for IS on reproductive efficiency, perinatal outcome and growth trajectory	Pregnancy rates and number of pups/pregnancy lower in Tac groups but no difference in number of live pups, neonatal deaths or birth weights	Díaz-García *et al.* (2014)

Live birth feasible post-UTx in a large animal species	Autologous sheep	Live birth	Live lambs delivered in 3/5 sheep	Wranning *et al.* (2010)

Pregnancy possible post-allogeneic UTx in a large animal	Allogeneic sheep	Live birth	Live lamb delivered in 1/5 sheep	Ramirez *et al.* (2011)

A uterus of similar size to human is tolerant to 24 h CIT	Autologous sheep	Tolerance of graft to 24 h CIT	3/4 uteri viable 8 days post-transplantation	Tricard *et al.* (2017)

Live birth after autologous UTx in a non-human primate is feasible	Autologous non-human primate	Live birth	1/2 animals achieved pregnancy with delivery via Caesarean section	Mihara *et al.* (2012)

Allogeneic live donor UTx in a non-human primate is possible but further research is needed to optimize IS protocols. Cervical biopsies are sufficient to diagnose rejection.	Allogeneic non-human primate	Effect of IS induction with ATG followed by maintenance IS with Tac +/− MMF and corticosteroids on resumption of cyclicity and graft acceptance	Menses occurred in 5/10 animals immunosuppressed with ATG followed by Tac + MMF + corticosteroids but normal microscopic graft appearance in only 1/10 animals; cervical biopsies monitored rejection	Johannesson *et al.* (2013)

Induction IS is required and maintenance IS should involve a combination of IS drugs	Allogeneic non-human primate	Feasibility and safety of using a DD model after use of varying induction and maintenance IS regimens	1/6 transplanted animals resumed normal reproductive activities with good physical health post treatment of rejections with ATG and shift from oral to intramuscular administration of Tac	Tryphonopoulos *et al.* (2014)

Live birth after allogeneic UTx in a non-human primate is feasible	Allogeneic non-human primate	Reproductive function and live birth	Cyclicity resumed in 5/6 transplanted animals and live birth occurred in 1/6 after extensive IS modifications	Kisu *et al.* (2020)

ATG, antithymocyte globulin; CIT, cold ischemic time; DD, deceased donor; IS, immunosuppression; MMF, mycophenolate mofetil; Tac, tacrolimus; UTx, uterus transplantation.

### Rodent investigations

The first attempts to perform UTx were undertaken in mouse using the syngeneic model to avoid the need for immunosuppression (IS) and challenges of rejection. Following demonstration of pregnancy post-transfer of blastocysts (Racho El-Akouri *et al.*, 2002), the surgical approach was modified by creating a cervical-cutaneous stoma, to allow drainage of mucous (Racho El-Akouri *et al.*, 2003a) and pregnancies with live births occurred, respectively, in nine native and eight transplanted uteri, with no alteration in implantation/miscarriage rates in the transplanted *versus* the native uterus and with normal growth trajectories to 8 weeks post-UTx; birthweights of second-generation offspring were normal. The important issue of duration of tissue viability between procurement and transplantation was subsequently addressed by comparing the effects of 24 h versus 48 h cold ischemic time (CIT) on fertility. Uteri preserved with a CIT of 24 h, but not 48 h, supported normal pregnancies and resulted in offspring with normal growth-trajectories during the 8 weeks post-natal course (Racho El-Akouri *et al.*, 2003b). These findings indicated uterine tolerance to extended CIT to 24 h and that any reperfusion-associated tissue damage is reversible. The final murine studies investigated any dose-dependent effect of the calcineurin inhibitor (CIN), cyclosporine, on intact females (without UTx) and offspring (Groth *et al.*, 2010). High-dose cyclosporine during mating and throughout pregnancy resulted in a 25% decreased implantation rate and a 2-fold increased miscarriage rate, although there was no effect on fertility of the second generation. Collectively, the results showed that exposure to high levels of cyclosporine during pregnancy negatively affects reproductive performance and pregnancy outcome.

Following demonstration that UTx in a syngeneic animal model resulted in live birth, attention was given to investigate whether pregnancy is achievable in an allogeneic model with use of IS, as this would mimic the approach used in clinical UTx. Using two strains of rat, one for the donor and the other for recipient (Diaz-Garcia *et al.*, 2010), and tacrolimus (Tac) for IS, pregnancy occurred after natural mating. In a more detailed follow-up study (Díaz-García *et al.*, 2014) it was found that pregnancy rates (number of pregnancies/mated rat) were higher in the control groups (80% and 70% in sham-operated-with-Tac group and sham-operated-non-Tac group, respectively) compared with the UTx-Tac group (50%). Birth weights and growth trajectories of offspring were similar among the three groups.

### Domestic species investigations

Although results of the above rodent studies suggested feasibility of UTx as a future treatment for clinical infertility, investigations were needed with large animals with vascular dimensions and gestational lengths more like those of the human. The sheep and pig have been systematically studied in UTx research. Although considerable contributions were achieved in some allogenic porcine UTx investigations (Avison *et al.*, 2009; Zhang *et al.*, 2018), we have chosen to focus here on the more extensive ovine studies.

In efforts to assess the feasibility of UTx in a large species, a method for autologous UTx in the sheep was first developed involving excision of one uterine horn and unilateral anastomoses (Dahm-Kahler *et al.*, 2008; Wranning *et al.*, 2008b). This was followed by demonstration of its success, with live births achieved in three of five mated animals post-UTx (Wranning *et al.*, 2010). A follow-on study then investigated fertility after allogeneic sheep UTx using cyclosporine IS (Ramirez *et al.*, 2011). Two ewes became pregnant after ET to five animals. Fetal demise occurred in one but a lamb with cardiac stability was delivered at 135 days of gestation from the other sheep (Ramirez *et al.*, 2011). This event was a milestone toward development of clinical UTx because it demonstrated normal progression of pregnancy in an allogeneic uterine graft with a long pregnancy time and of a uterine size similar to the human. A small number of additional UTx studies in the ewe were performed with significance for human UTx. One of these focused on studying an optimum IS protocol (Gauthier *et al.*, 2011) while another found tolerability of a large-sized uterine graft to ischemia-reperfusion after 24 h of CIT (Tricard *et al.*, 2017). Studies on UTx with the sheep have continued at several centers, both for surgical team training in preparation for human UTx (Solomonov *et al.*, 2017), in the event that cadaveric training is not feasible, and to test new UTx techniques, such as laparoscopic uterus procurement (Sánchez-Margallo *et al.*, 2019), anastomosis techniques (Arantes *et al.*, 2020; Maraschio *et al.*, 2021), and biomarkers for ischemia-reperfusion (Carbonnel *et al.*, 2021).

### Non-human primate investigations

Although further surgical skills were acquired from studies in domestic species, investigations on non-human primates (NHPs) were considered necessary. Not only do these animals have reproductive physiology and anatomy similar to that of human, but it was considered important to follow the first ethical guidelines concerning UTx, which state that ’uterine transplantation, which may reach human clinical experimentation stage, should only occur after significant and adequate research in appropriate large animal models, including primates’ (Milliez, 2009). However, NHP studies on pregnancy after allogeneic UTx were not performed before the first human UTx trial (Brännström *et al.*, 2014). The two NHP species primarily used in UTx research are the baboon (*Papio anubis* and *Papio hamadryas*) and cynomolgus macaque (*Macaca fascicularis*). The advantage of the baboon is its larger size (10–20 kg) versus the smaller cynomolgus macaque (3–4 kg).

The early NHP studies used autologous models to investigate the efficacy of several surgical approaches for UTx (baboon; Enskog *et al.*, 2010); and cynomolgus macaque (Mihara *et al.*, 2012). Following end-to-end anastomoses on the external iliacs of uterine arteries, one deep uterine vein, and one ovarian vein in two transplanted cynomolgus macaques, pregnancy after spontaneous mating occurred and with Caesarean section of a liveborn (Mihara *et al.*, 2012). This was the first live birth after UTx in a primate species, albeit in an autologous model. Investigating IS protocols in NHPs was considered essential in the continued development of UTx as a clinical procedure. Although allogeneic LD UTx in the baboon showed that an ‘IS-heavy’ protocol with induction therapy (antithymocyte globulin (ATG)) followed by triple IS (Brännström *et al.*, 2022a) resulted in resumption of hormonal cyclicity in five of the ten animals, normal uterine microscopic appearance was seen in only one baboon after 3 months (Johannesson *et al.*, 2013). However, an important finding was that cervical biopsies seemed sufficient to diagnose rejection, which led to development of a scoring system for uterine rejection (Johannesson *et al.*, 2013), later modified and now routinely used in human UTx (Mölne *et al.*, 2017). The poor outcomes in the above IS study (Johannesson *et al.*, 2013) indicated that further studies were warranted in a NHP model. Consequently, a follow-up study, also in baboon, investigated induction IS with ATG and high doses of corticosteroids, followed by maintenance IS with Tac and corticosteroids (Tryphonopoulos *et al.*, 2014). Rejection episodes were treated by ATG and a shift from oral to i.m. administration of Tac. However, only one of the four animals resumed good physical health and had an extended period of normal reproductive activities with a graft of normal physiology with hysteroscopy at 4 months. Collectively, the findings of this allogeneic UTx study further emphasized the need for induction IS and that maintenance IS, with a combination IS, should be used.

The ultimate goal of UTx is a live birth. As elaborated above, only one NHP live birth, which was achieved after auto-transplantation, had been reported (Mihara *et al.*, 2012) before human UTx trials began. It is therefore worth noting the only other NHP study in which a live birth was achieved, because this occurred in the allogeneic model. In an extensive study by the Japanese group, using an even stronger IS protocol than they used previously (Kisu *et al.*, 2019), two transplanted cynomolgus macaque females underwent ET at 1–1.5 years after UTx, one of which had 10 ETs but no pregnancy. Although the second animal achieved early pregnancies, miscarriages occurred on initial ETs. On the fifth ET, successful pregnancy occurred, and elective Caesarean section was performed at full-term. A healthy offspring was delivered (Kisu *et al.*, 2020).

## Clinical flow of human uterus transplantation

**Figure 2.**
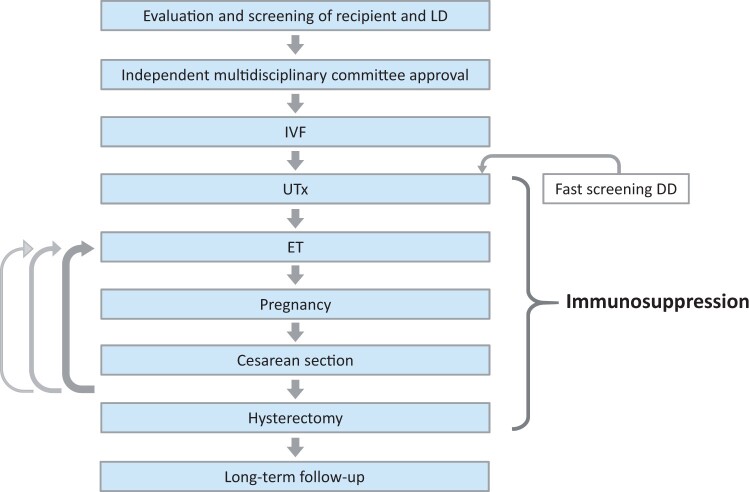
**Flowchart of uterus transplantation in human**. The arrows on the left indicate that pregnancy and delivery (by Caesarean section) can be repeated several times. LD, live donor; DD, deceased donor; ET, embryo transfer; UTx, uterus transplantation.

The steps of a typical human UTx procedure are shown in Fig. 2. After extensive laboratory work-up, imaging, and psychological screening of a potential recipient, her committed LD will also undergo screening. An independent multidisciplinary committee should then scrutinize all screening details before final approval of the recipient and the LD to proceed with IVF and surgeries. In the event of a planned DD procedure, a rapid and less extensive donor screening will generally be performed, ideally within 24 h of the UTx procedure. From the day of UTx, the recipient will be on IS and the first ET will generally be performed within 3–12 months of the transplantation. Pregnancy can be monitored normally and the mode of delivery should be Caesarean section. If more than one pregnancy is desired, this can be recommended providing there are no medical contraindications. To minimize long-term side effects of IS, hysterectomy should be performed after birth of the desired number of children or after excessive repeated implantation failure or miscarriages. The recipient, LD and children born should be followed for psychological and medical health for several years after the procedure.

## Surgery

### Surgical techniques for deceased donor hysterectomy

Hysterectomy in DDs is through a full midline incision. The ureters are transected above the uterus and vascular pedicles, including the internal iliacs plus uterine vessels, are dissected caudally from common iliacs. All branches from the internal iliacs, except uterine vessels, are ligated and divided. The rectovaginal space is then opened, and the ureters are transected near the bladder. After the cervix and upper vagina are freed from the bladder, full uterine dissection occurs. Procurement of abdominal organs generally takes place before the uterus is flushed via femoral catheters (D’Amico *et al.*, 2021) although a technique of performing the DD hysterectomy before procurement of abdominal organs has been described (Testa *et al.*, 2018a).

### Surgical techniques for live donor hysterectomy

**Figure 3.**
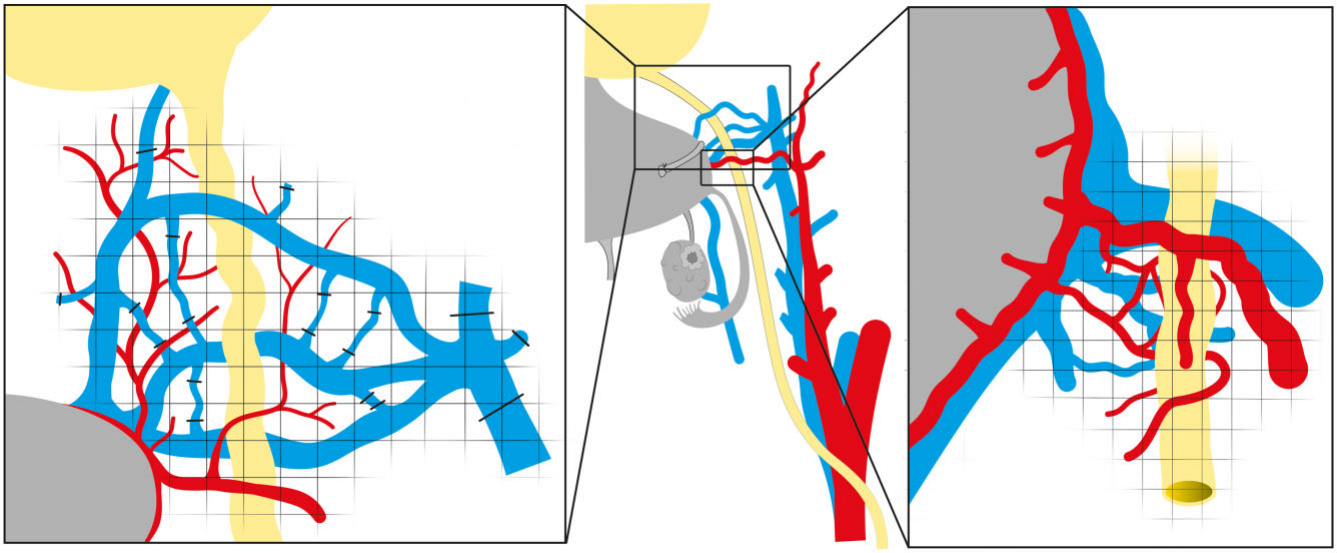
**Schematic drawing of the operating field of the donor’s right pelvic side at hysterectomy**. The left panel is a close up of the surgical field at the distal aspect of the ureter. The middle panel is an overview of the entire surgical field. The right panel is a close up of the surgical field of the ureteric tunnel. In all panels, arteries are red, veins are blue, ureter, and bladder are yellow, and uterus is grey. The squared grid illustrates those areas covered in connective tissue.

**Figure 4.**
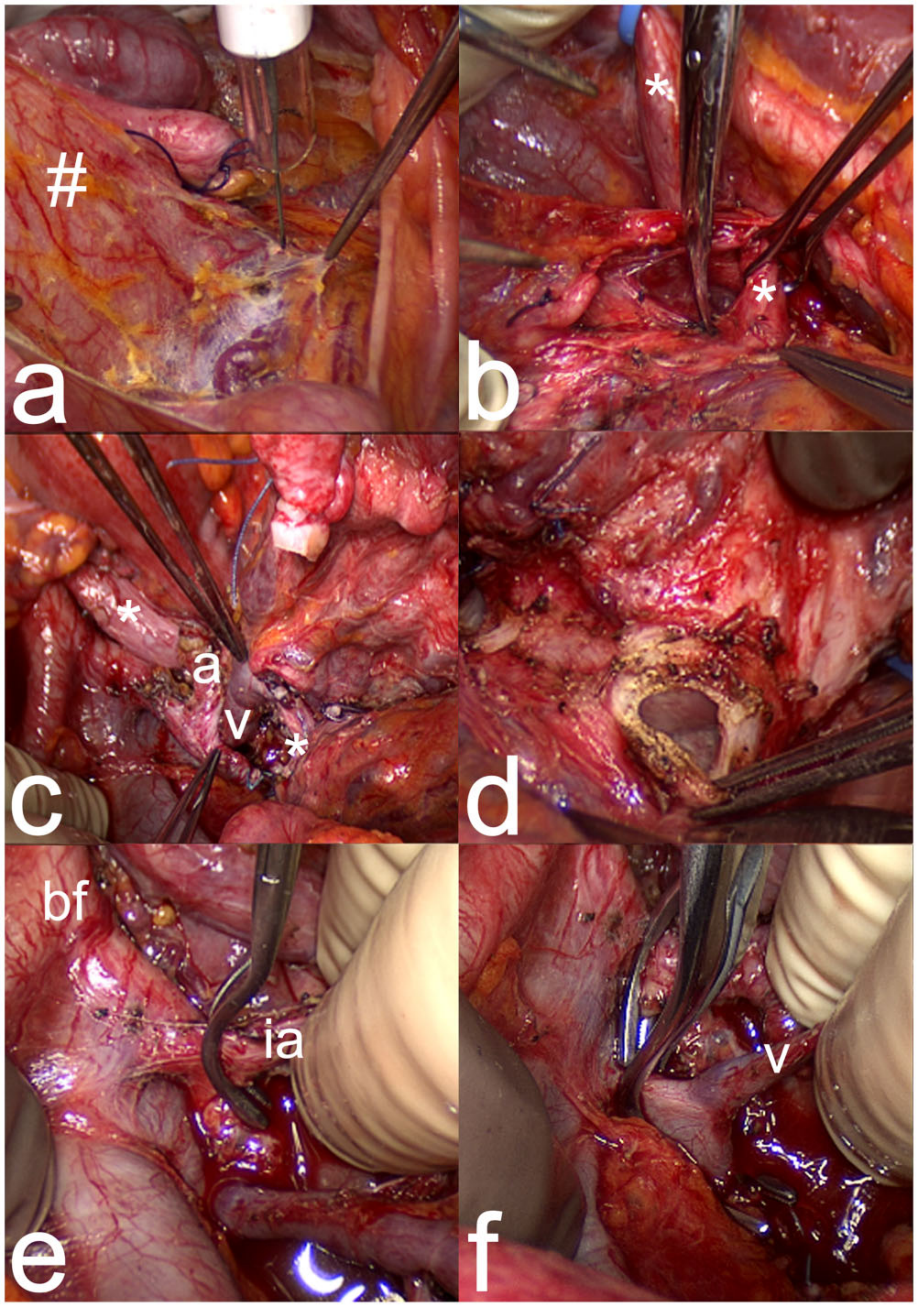
**Photographs showing specific steps in live donor hysterectomy by laparotomy**. (**a**) A large peritoneal flap (#) is dissected from the bladder dome to be included in the graft after the round ligaments have been divided and tagged. (**b)** The right ureter (*) is dissected free. (**c**) Image showing completed dissection on the right pelvic sidewall of the ureter (*), a deep uterine vein (v), and the uterine artery (a) extending from the anterior internal iliac artery. (**d**) The vagina of the donor is transected as the last step before vascular clamping with transection of vessels and extraction of the organ. (**e**) The right anterior division of the internal iliac artery (ia) is clamped, leaving the major posterior branch, before transection of the major vessels and removal of the uterine graft from the donor. The bifurcation (bf) of the right common iliac artery into the external and internal iliac arteries is seen to the left. (**f**) A vascular clamp is placed over the left internal iliac vein, to include a segment of this vein together with the deep uterine vein (v) of the graft.

**Figure 5.**
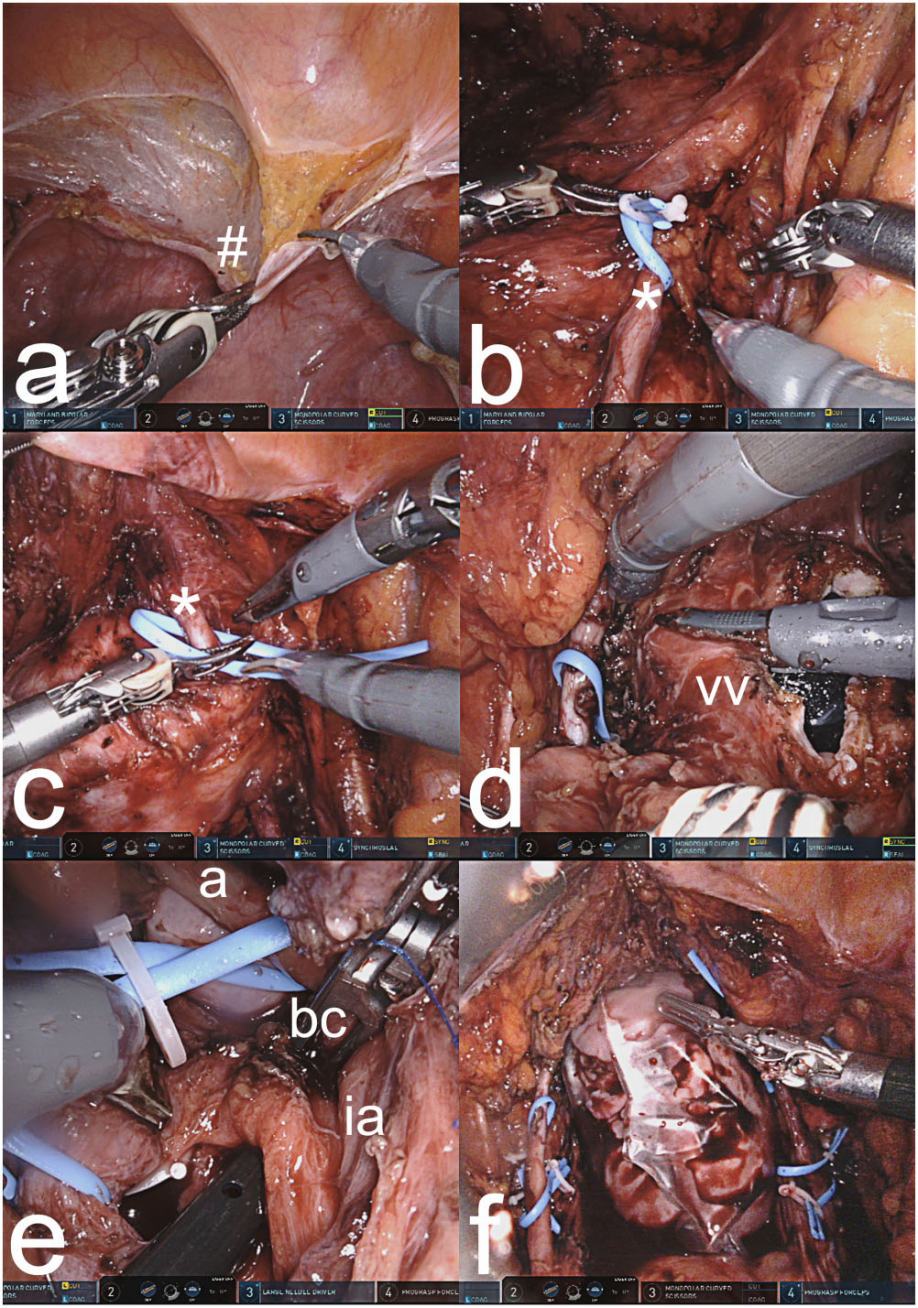
**Photographs showing specific steps in live donor hysterectomy by robotics**. (**a**) The peritoneal flap (#), to be included in the graft, is dissected off from the bladder dome. (**b**) Image showing dissection of the right ureteric tunnel with the proximal ureter (*) in a blue rubber band. (**c**) Dissection of the distal portion of the right ureter (*) before the inlet to the bladder. (**d**) The vaginal vault (vv) is opened. (**e**) A bulldog clamp (bc) after being positioned on a major branch of the right anterior branch of the internal iliac artery (ia) before transection of major graft vessels. The upper rubber band is around the arterial trunk (a) leading to the uterine artery. The lower rubber band is placed around the ureter. (**f**) The uterus is placed in a laparoscopic bag and is extracted through the vagina.

LD hysterectomy, clearly more complicated than DD hysterectomy, can be performed by laparotomy or robotics. With laparotomy, a sub-umbilical midline incision is used while in robotics, typically five working ports are used, three for robotics and two for laparoscopy. Regardless of approach, surgical duration is around 10 h and involves a similar sequence of surgical sub-steps in the pelvis (Fig. 3), as described previously (Brännström *et al.*, 2014, 2020c). Photographs of the LD hysterectomy by laparotomy and robotics are shown in Figs 4 and 5, respectively. The surgery is first directed to transect round ligaments and open the vesicovaginal space. Dissections of two areas of the ureter are especially demanding, namely the ureteric tunnel and the distal aspect of the ureter, which is the area from the tunnel-outlet to the bladder. In the ureteric tunnel, there will typically be an over-riding uterine artery and under- or over-riding deep uterine vein(s). The tunnel is covered by connective tissue with several small arteries and veins, and these vessels need to be divided. At completion of the tunnel dissection, the ureter should be fully freed, enabling identification of the ureter at the outlet. At the distal aspect, the ureter rides closely to the deep uterine veins and with several smaller vessels in the area, as well as covered by connective tissue. The large vessels are firmly attached to the ureter and the cervix. Typically, one or two deep uterine/inferior uterine veins are used in a uterine graft and several small vessel branches must be divided. The bilateral vascular pedicles on the arterial side (uterine artery with anterior portion of internal iliac artery) and the venous side (deep uterine/inferior uterine veins(s) with segment of internal iliac vein) are then dissected with ligation and transection of branches (Brännström *et al.*, 2020c). In cases with thin deep uterine veins(s) insufficient for venous outflow, the uterine branch of the utero-ovarian vein/superior uterine vein is dissected. Before procurement, the oviducts, the utero-ovarian ligaments, and the sacro-uterine ligaments are divided. The vagina is transected 2 cm below the cervix. The vascular pedicles are clamped and transected with back-table flushing and cooling.

### Surgical techniques for transplantation in the recipient

**Figure 6.**
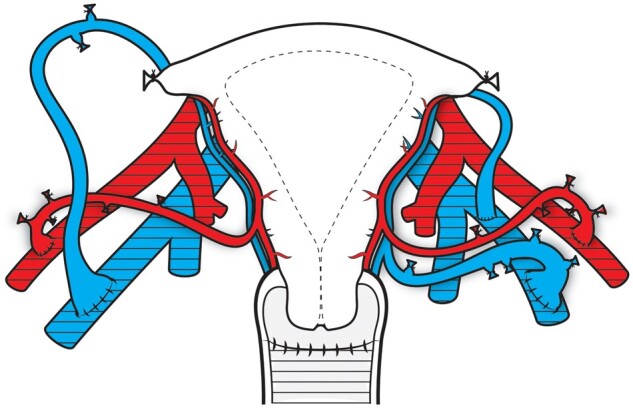
**Schematic drawing of the vascular and vaginal anastomoses in the recipient**. The tissues of the recipient are covered by a lined grid. The anterior portions of the internal iliac arteries are anastomosed end-to-side to the external iliac arteries on both sides. On the recipient’s left side, one deep uterine vein and the uterine branch of the utero-ovarian vein are anastomosed end-to-side to the external iliac vein. On the recipient’s right side, the utero-ovarian vein is anastomosed end-to-side to the external iliac vein.

**Figure 7.**
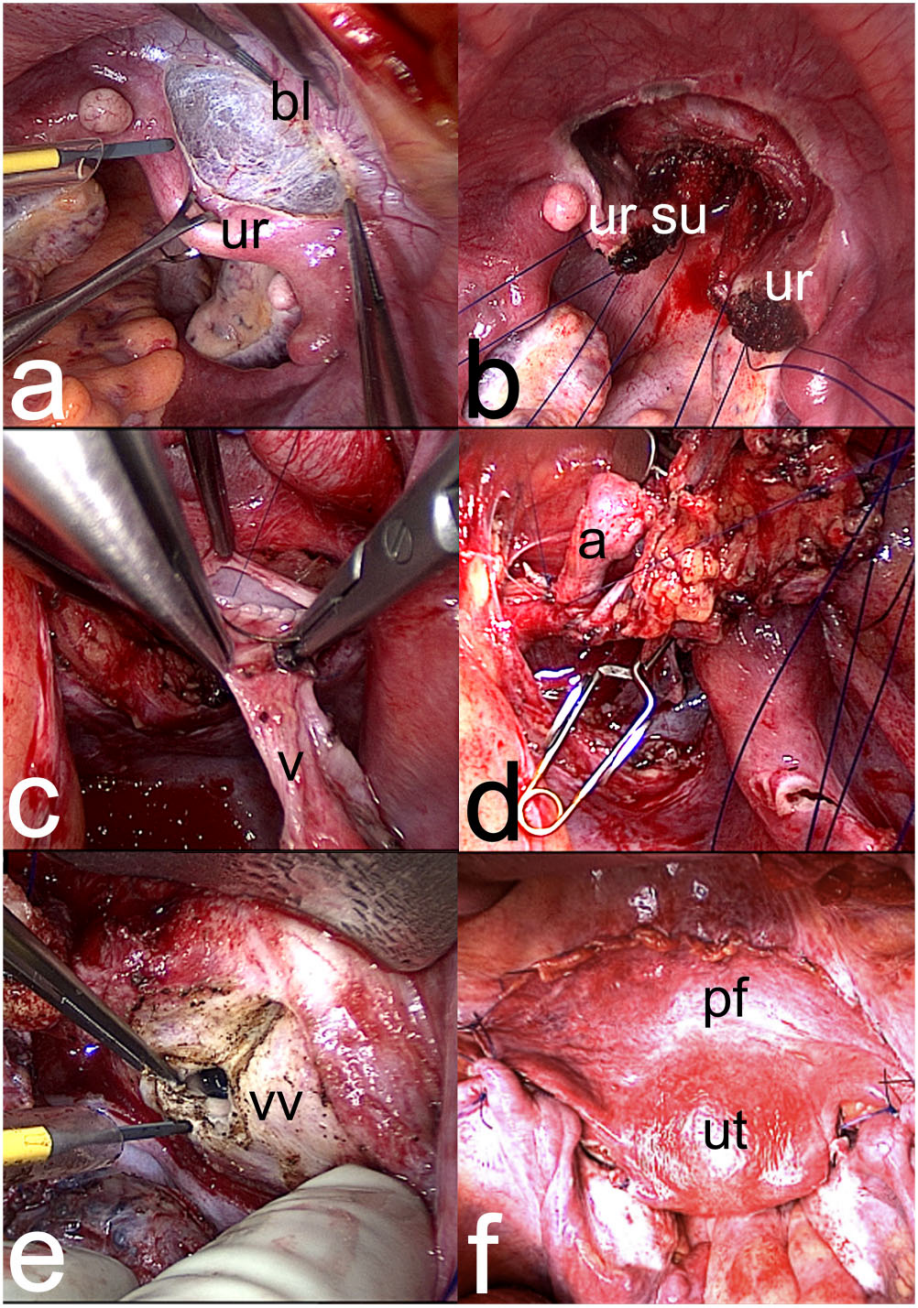
**Photographs showing specific steps in recipient surgery at uterus transplantation**. (**a**) The midline uterine rudiment (ur) in a patient with Mayer–Rokitansky–Küster–Hauser syndrome is lifted by forceps with simultaneous dissection to separate the bladder (bl) from the rudiment and the vaginal vault. (**b**) Image showing the uterine rudiment after cleavage in the midline down to the vaginal vault, with fixation sutures placed through the sacrouterine ligaments (su) and through both sides of the cleaved uterine rudiment (ur). (**c**) Image showing end-to-end vascular anastomose under completion between a deep uterine vein (v) of the graft and the external iliac vein of the recipient. (**d**) An end-to-end vascular anastomose is completed between the artery (a) of the graft and the external iliac artery of the recipient. A bulldog clamp is placed proximally to the completed venous anastomosis. (**e**) The vaginal vault (vv) is opened longitudinally by monopolar diathermy. (f) The grafted uterus (ut) is seen after completed transplantation, with a peritoneal flap (pf) overlaying the recipient’s bladder.

The recipient surgery is similar in DD and LD UTx. The duration of the laparotomy approach (Brännström *et al.*, 2014), which has been used in all published cases, is reported as 2–6 h in 73% of cases (Brännström *et al.*, 2023), which is considerably shorter than for LD donor hysterectomy. Recipient surgery by robotics has recently been performed in one UTx case (N. Kvarnström, personal communication), with similar steps as by laparotomy, but considerably longer duration. The vascular and vaginal anastomoses in the recipient are schematically shown in Fig. 6 and with photographs of recipient surgery in Fig. 7. The surgery starts with clearance of the vaginal vault from the bladder and the external iliac vessels. In a woman with Mayer–Rokitansky–Küster–Hauser syndrome (MRKHs) (Herlin *et al.*, 2020), the rudimentary uterus in the midline is cleaved to the vault level. The graft is then lifted into the pelvis to perform end-to-side anastomoses of the uterine vessels to the external iliac vessels (Brännström *et al.*, 2020a). The vault is opened and vaginal–vaginal anastomosis is accomplished. Fixation sutures connect the round and the sacrouterine ligaments.

### Uterus transplantation cases and trials—surgical success and postoperative complications

The first LD and DD UTx procedures were performed in 2000 (Fageeh *et al.*, 2002) and 2011 (Ozkan *et al.*, 2013), respectively (Fig. 1). The first clinical UTx trial was an observational study, including nine laparotomy LD UTx procedures in Sweden in 2012–2013 (Brännström *et al.*, 2014). Typically, it will take at least 3–5 years after the last UTx procedure of a trial until the reproductive window of the participants is closed by graft removal. The UTx study from 2012 to 2013 is so far the only UTx trial that has reported complete reproductive and obstetrical data (Brännström *et al.*, 2022a).

In the present section, we incorporate data from all published UTx cases (n = 71), some presented as single cases and the majority within trials. The publications of UTx trials include material from the first Swedish trial with transplantations from 2012 (nine laparotomy LD-UTx procedures) (Brännström *et al.*, 2014), in the USA the Dallas trial with transplantations from 2016 (13 laparotomy LD-UTx, five robotic LD-UTx, and two DD-UTx procedures) (Testa *et al.*, 2020), in the USA the Cleveland trial with transplantations from 2016 (eight DD-UTx procedures) (Richards *et al.*, 2021), the Czech trial with transplantations from 2016 (five DD-UTx and five laparotomy LD-UTx procedures) (Fronek *et al.*, 2021), the German trial with transplantations from 2016 (four laparotomy LD-UTx procedures) (Brucker *et al.*, 2020), the Indian trial with transplantations from 2017 (four laparoscopy LD-UTx procedures) (Puntambekar *et al.*, 2018, 2019) and the second Swedish trial with transplantations from 2017 (eight robotic LD-UTx procedures) (Brännström *et al.*, 2020a,c). Publications of single laparotomy LD-UTx procedures are from Saudi Arabia (Fageeh *et al.*, 2002) and Lebanon (Akouri *et al.*, 2020). Reports of single robotic LD-UTx procedures are from China (Wei *et al.*, 2017), Spain (Carmona *et al.*, 2021), France (Ayoubi *et al.*, 2022), and Brazil (Vieira *et al.*, 2021). Publications of single DD-UTx procedures are from Turkey (Ozkan *et al.*, 2013) and Brazil (Ejzenberg *et al.*, 2019).

The surgical success of UTx, defined as a case resulting in normal blood flow post-transplantation with regular menstruations, can be assessed after around 4 months. The total surgical success (Table 2) in laparotomy LD-UTx (24/33; 73%) was in the same range as DD-UTx (12/17; 71%), which is lower than in laparoscopy LD-UTx (4/4; 100%), and robotic LD-UTx (15/17; 88%). However, laparoscopic and robotic LD-UTx were introduced >4 years after laparotomy LD-UTx and the knowledge acquired from laparotomy cases was most likely of benefit when minimal invasive surgery (MIS) was introduced in LD-UTx. Overall, surgical success was achieved in 55 of the 71 cases (77%).

**Table 2.** Uterus transplantation trials and cases, with rates of surgical success and major post-operative complications of live donors.

Trials (city)*	From year	Type of case	# cases	# Surgical successes	# LD cases	# LD post-op complications	Reference
Sweden	2012	Laparotomy LD	9	7 (78%)	9	1 (11%)	Brännström *et al.* (2014)
USA (Cleveland)	2016	DD	8	6 (75%)	n/a	n/a	Richards *et al.* (2021)
Czech Republic	2016	Laparotomy LD	5	4 (80%)	5	2 (40%)	Fronek *et al.* (2021)
		DD	5	3 (60%)	n/a	n/a	
USA (Dallas)	2016	Laparotomy LD	13	8 (62%)	13	2 (15%)	Testa *et al.* (2020)
		Robotic LD	5	5 (100%)	5	2 (40%)	
		DD	2	1 (50%)	n/a	n/a	
Germany	2016	Laparotomy LD	4	4 (100%)	4	0 (0%)	Brucker *et al.* (2020)
India	2017	Laparoscopy LD	4	4 (100%)	4	0 (0%)	Puntambekar *et al.* (2018, 2019)
Sweden	2017	Robotic LD	8	6 (75%)	8	1 (13%)	Brännström *et al.* (2020a,c)

Single cases (city)*	**Year**	**Type of case**	**# cases**	**# Surgical successes**	**# LD cases**	**# LD post-op complications**	**Reference**

Saudi Arabia	2000	Laparotomy LD	1	0 (0%)	1	0 (0%)	Fageeh *et al.* (2002)
Turkey	2011	DD	1	1 (100%)	n/a	n/a	Ozkan *et al.* (2013)
China	2015	Robotic LD	1	1 (100%)	1	0 (0%)	Wei *et al.* (2017)
Brazil (Sao Paulo)	2016	DD	1	1 (100%)	n/a	n/a	Ejzenberg *et al.* (2019)
Lebanon	2018	Laparotomy LD	1	1 (100%)	1	0 (0%)	Akouri *et al.* (2020)
France	2019	Robotic LD	1	1 (100%)	1	1 (100%)	Ayoubi *et al.* (2022)
Spain	2020	Robotic LD	1	1 (100%)	1	0 (0%)	Carmona *et al.* (2021)
Brazil (Barretos)	2021	Robotic LD	1	1 (100%)	1	0 (0%)	Vieira *et al.* (2021)

**All cases**		**Type of case**	**# cases**	**# Surgical successes**	**# LD cases**	**# LD post-op complications**	

		Laparotomy LD	33	24 (73%)	33	5 (15%)	
		Robotic LD	17	15 (88%)	17	4 (24%)	
		Laparoscopy LD	4	4 (100%)	4	0 (0%)	
		DD	17	12 (71%)	n/a	n/a	

**Totals**		**All types**	**71**	**55 (77%)**	**54**	**9 (17%)**	

* City indicated if more than one city in a country. LD, live donor; DD, deceased donor.

Postoperative complications have been reported according to the Clavien–Dindo (CD) system (Dindo *et al.*, 2004). Major complications are those of CD-III, requiring radiological, endoscopic or surgical intervention and of CD-IV, with organ dysfunction. Graft failure and transplantectomy within the initial months are classified as CD-III.

The overall rate of major postoperative LD complications (>CD-II) was 9/54 (17%), the majority of which were related to the urinary system and involved hydronephrosis, ureteric fistula and hypotonic bladder (Table 3). The urinary tract complications most likely result from challenges in dissection at the intersections of the ureters and uterine vessels, with lacerations and thermal injuries. Proposed strategies to decrease ureteric lesions include use of ureteric stents, avoidance of diathermy close to the ureter, and use of indocyanine green to identify ureters and vessels (Johannesson *et al.*, 2021a). Efforts to reduce surgical duration by alternatives to deep uterine veins as venous outflows have been made, showing shortened duration by using the ovarian branches of the utero-ovarian veins with anastomosis to the external iliac veins (Testa *et al.*, 2020). Of note, this does not necessitate oophorectomy.

**Table 3.** Reported complications (>Grade 2 Clavien–Dindo) in live donors and recipients.

Donor	Complication
Ureter	Laceration
	Uretero-vaginal fistula
	Blood clot
	Hydronephrosis
Bladder	Hypotonia
Vagina	Cuff dehiscence
Infection	Pyelonephritis
Other	Fecal impaction
	Hemorrhage

**Recipient**	**Complication**

Transplantectomy before childbirth	Thrombosis
	Hypoperfusion
	Intra-uterine infection
	Post-transplantation lymphoproliferative disorder
	Repeated miscarriages/implantation failures
	Irreversible endometrial damage
	Rejection
Bladder	Vesico-vaginal fistula
Vagina	Vaginal stricture
Other	Per-operative hemorrhage
	Midline incisional hernia

The most common complication in recipients has been early graft failure, which is equal to cases with no surgical success. Graft failures within the first months post-UTx occurred in 23% of the cases, the rate being 29% after DD-UTx, 27% after laparotomy LD-UTx, 12% after robotic LD UTx, and 0% after laparoscopic UTx (Table 2). Major reasons for graft failure with subsequent transplantectomy before childbirth are thrombosis of graft vessels or hypoperfusion, resulting in uterine necrosis (Table 3) (Brännström *et al.*, 2023). Likely causes are uterine vessels of too low caliber or atherosclerosis. Thus, as highlighted by two UTx procedures that had to be interrupted after LD hysterectomy due to discovery of poor uterine artery quality at back-table preparation (Brucker *et al.*, 2018; Fronek *et al.*, 2021), preoperative imaging of recipient vasculature should be performed to exclude cases at high risk for low blood flow (Leonhardt *et al.*, 2022). Apart from ischemia-related issues, occasional graft removals within the first 8 months after UTx have occurred because of intrauterine-infection, irreversible endometrial damage, and post-transplant lymphoproliferative disorder (Brännström *et al.*, 2023). There is an increased risk of recipient hemorrhage after a DD UTx because multiple venous branches may not have been adequately ligated at the fast procurement from a multiorgan donor (Ejzenberg *et al.*, 2019). To avoid this, meticulous dissection with identification of leakage points should be performed at back-table (Testa *et al.*, 2017). Another complication among recipients, which typically occurs several months after UTx, is vaginal stricture over the suture line. This may cause problems with rejection monitoring by cervical biopsies and ET. Data from the USA showed vaginal strictures in 72% of recipients, with half of them treated by nonsurgical dilatation and the rest by surgery (Johannesson *et al.*, 2022).

## IVF in patients undergoing uterus transplantation

Because current UTx does not involve Fallopian tube transplantation, success of the procedure depends on IVF to create embryos for later transfer. Owing to the temporary nature of the uterine graft, IVF must be optimized for maximal efficiency and safety.

To date, almost all UTx patients had MRKHs and were young with 94% MRKHs and mean age of 31 years in the US cases (Johannesson *et al.*, 2022) and 98% MRKHs and mean age 29 years in international cases of the ISUTx registry (Brännström *et al.*, 2023). Although ovarian reserve is generally good in this age group, special issues must be considered when performing IVF in women with MRKHs, including possible susceptibility to vaginal/cervical infections and secondary miscarriage in MRKHs women with neovagina of a type that is not from dilation, and thereby with a non-physiological vaginal mucosa. Also, women with type B MRKHs (with urinary tract malformation) seem to have lower levels of anti-Müllerian hormone, lower antral follicle counts, and a decreased response to gonadotrophins, as compared to type A MRKHs (Raziel *et al.*, 2012).

### Infectious disease screening before IVF

Most IVF programs require screening for serious viral infections and for syphilis. Such screening is especially important for immunosuppressed UTx recipients so as to minimize risk of infection during pregnancy.

### Gonadotrophin stimulation and oocyte retrieval

Candidates for UTx must be excellent candidates for IVF and so should be screened for normal to high ovarian reserve. Although performing IVF before UTx has undeniable benefits, additional IVF cycles have been performed after UTx in many trials because of exhaustion of pre-UTx embryos or couple separation and patient wish to attempt pregnancy with either a new partner or donor sperm. These post-UTx IVF cycles have been without any complications and have resulted in several live births (Brucker *et al.*, 2020; Brännström *et al.*, 2022a).

In ongoing UTx trials, both long protocols with GnRH agonist and hCG trigger, and short protocols with GnRH antagonists and GnRH-agonist trigger, have been used; however, the majority uses the latter to minimize risk of ovarian hyperstimulation syndrome, which has occurred in one UTx patient (Brännström *et al.*, 2022a). Monitoring follicular growth by transvaginal ultrasound may be impossible in those women with MRKHs with ovaries localized in a more cranial position than usual. In such cases, measurement of estradiol is a useful marker for follicular growth and development.

Oocyte retrieval in women with MRKHs is typically performed transvaginally or transabdominally depending on individual anatomy (Chmel *et al.*, 2020). In the original Swedish UTx trial, five of nine patients had transabdominal retrieval (Brännström *et al.*, 2022a).

### Should oocytes or embryos be frozen and how many?

In all currently reported cases, embryos rather than oocytes have been frozen. However, cryopreserving some oocytes may be prudent in case the couple separates before ET. The number frozen should consider a likely attrition of 10–15% post-thaw, and that the implantation rate of embryos from vitrified oocytes is typically lower than from fresh oocytes (Jia and Sun, 2021). Additionally, wishes to have more than one child should be considered.

Most teams currently require banking of 5–10 embryos before UTx. However, both embryo stage and quality should be considered (Glujovsky *et al.*, 2022). In the first completed UTx trial, live birth rates per ET with cleavage stage *versus* blastocyst embryos were 12.5% and 45.4%, respectively (Brännström *et al.*, 2022a).

### Should preimplantation genetic testing for aneuploidy be used for all patients undergoing uterus transplantation?

There is ongoing debate regarding whether and to whom preimplantation genetic testing for aneuploidy (PGT-A) should be used for prospective UTx recipients (Chattopadhyay *et al.*, 2021). One UTx group transferred single good quality, expanded, euploid blastocysts after PGT-A in a cohort of 14 patients and reported a high rate of clinical pregnancy per ET (Putman *et al.*, 2021). Potential benefits of PGT-A include reductions in time to pregnancy (Neal *et al.*, 2018), costs (Lee *et al.*, 2018; Neal *et al.*, 2018), and risk of miscarriage (Scott *et al.*, 2013; Rubio *et al.*, 2019). However, arguments against use of PGT-A include possible adverse obstetrical outcomes caused by the biopsy procedure (Hou *et al.*, 2021; Makhijani *et al.*, 2021), questionable efficacy of PGT-A to improve live birth rates (Munne *et al.*, 2019; Ozgur *et al.*, 2019), possible need to undergo additional cycles to obtain enough euploid embryos (Chattopadhyay *et al.*, 2021) and additional costs (Table 4). The nuances associated with these opposing arguments indicate that adequate counseling is paramount and the decision to proceed with PGT-A should be individualized.

**Table 4.** The pros and cons of preimplantation genetic testing for aneuploidy in the uterus transplantation population.

Arguments in support of using PGT-A	**Evidence in favor of using PGT-A** *	Favorable outcome
Reduced time to pregnancy	Neal *et al.* (2018, p. 110)	Reduced duration of exposure to IS medications.
	Lee *et al.* (2018)	
Reduced cost	Neal *et al.* (2018)	Reduced emotional burden and potentially increased access to UTx treatment.
Reduced risk of miscarriage	Rubio *et al.* (2019)	Reduced time to a healthy pregnancy and reduced risks of infection.
	Scott *et al.* (2013)	
Reduced emotional burden	None	Reduced time to a healthy pregnancy.

**Arguments against using PGT-A**	**Evidence against using PGT-A** *	**Unfavorable outcome**

Questionable efficacy of PGT-A	Munne *et al.* (2019)	PGT-A may increase risk of discarding embryos due to: (i) false negative and false positive results; (ii) ‘no reads’ (i.e. no test result was obtained); (iii) mosaic results.
	Ozgur *et al.* (2019)	
Possibility of requiring additional oocyte retrievals	Reviewed by Chattopadhyay *et al.* (2021)	PGT-A is associated with some embryo attrition due to embryos unsuitable for biopsy or because some test as aneuploid or mosaic.
Increased risk of adverse obstetrical outcomes	Hou *et al.* (2021)	Day 5/Day 6 biopsy may be associated with an increased risk of very low birthweight and maternal hypertension.
Increased cost	Costs of PGT-A—FertilityIQ	The average cost of a PGT-A cycle is approximately $5000 in the USA and €3500 in Europe.

* Citations are examples of the available relevant literature. UTx, uterus transplantation; PGT-A, preimplantation genetic testing for aneuploidy; IS, immunosuppression.

### Embryo transfer

The ET procedure follows routine steps, although vaginal strictures can pose problems (Fronek *et al.*, 2021). Transfer of a single embryo should be compulsory for UTx recipients since transfer of multiple embryos markedly increases risks of a multiple pregnancy and obstetrical, neonatal and postnatal complications.

The original protocol by the Swedish group recommended a full year of close clinical observation post-transplantation before the first ET (Johannesson *et al.*, 2015), as recommended for women with solid organ transplants. This was an appropriately prudent approach, given the many unknowns at inception of the UTx field. However, recent reports of safe pregnancies and births following ETs at 4–6 months (Putman *et al.*, 2021) suggest that transfers early post-UTx are feasible provided an uneventful recovery, no recent rejection episode, and regular spontaneous or induced menstruations have occurred (Testa *et al.*, 2018b; Johannesson *et al.*, 2019). A shorter UTx-to-ET interval has psychological and physiological advantages (shorter time on IS) and represents a step forward for acceptance and success of UTx.

### Endometrial preparation

Endometrial preparation can be based on a natural cycle after spontaneous menstruation (Brännström *et al.*, 2022a). Alternatively, the endometrium is prepared with a programmed cycle, starting with exogenous estradiol for around 2 weeks, adding progesterone when the endometrium is >7 mm and synchronized with the ET, depending on day of embryo cryopreservation. There is no current preference or recommendation regarding which endometrial preparation is optimal after UTx; both approaches, as well as fresh ET, have produced live births. However, elevated estradiol levels typical of programmed cycles can affect CIN metabolism through direct inhibition of hepatic Cytochrome P450 3A (Migali and Tintillier, 2008), indicating that renal function should be monitored closely. Moreover, higher risks of pregnancy hypertension, postpartum hemorrhage, post-term birth, and macrosomia occur in programmed *versus* natural cycles (Ginström Ernstad *et al.*, 2019).

Patients with MRKHs may have altered vaginal epithelium, secondary to type of neovagina, which may influence resorption of vaginally administered progesterone (Chmel *et al.*, 2020) and bacterial colonization of the vagina. In the ISUTx registry report, 10% of patients had a vagina created by a split-skin graft (Brännström *et al.*, 2023). Our experience is that such grafting may lead to colonization by bacteria that are not part of the normal vaginal flora. This may be associated with implantation failures and repeated miscarriages (Brännström *et al.*, 2022a).

### Live birth rate and obstetric outcome

According to the ISUTx registry with 19 live births (Brännström *et al.*, 2023), the live birth rate/ET was 27.8% for cleavage stage embryos *versus* 40.0% for blastocysts, with an overall live birth rate/ET of 35.8%. The three major UTx centers in the USA reported 22 live births and a total live birth rate/ET of 35.6%. The median gestational age at birth was 36 weeks 6 days (range: 30 + 1 to 38 + 0) in reports from the USA (Johannesson *et al.*, 2022) and 35 completed weeks (range: 31–38) in the ISUTx registry (Brännström *et al.*, 2023). All deliveries were by Caesarean section. Almost half (47%) of US UTx neonates had at least 1 day in neonatal intensive care (Johannesson *et al.*, 2022) and in the original Swedish study, four of nine neonates developed respiratory distress syndrome (Brännström *et al.*, 2022a). These observations have led to extending the targeted delivery time from 35 weeks to more than 37 weeks in most centres.

## Long-term health outcome after uterus transplantation

Uterus transplantation may have life-long consequences for all four parties involved: the recipient, the recipient’s partner, the LD, and the children born. These consequences are possible despite UTx not being designed for life-long use. Indeed, transplantectomy should take place after all pregnancy attempts have been made and if the graft fails. In the original Swedish UTx study, uterine graft retention in those recipients with live birth(s) ranged from 1.8 to 5.9 years (Karlsson *et al.*, 2022).

To date, long-term follow-up data are only available from the Swedish laparotomy LD UTx trial (Brännström *et al.*, 2014, 2022a). Prospective data on the psychological and medical health of LDs, recipients, and recipient partners were collected from prior to UTx up to 4 years thereafter. Qualitative research data based on repeated recipient interviews have also been collected in the interval from UTx through 5 years (Järvholm *et al.*, 2020b).

### Live donor health outcomes

The extensive pelvic surgery of LDs may have long-term side effects, and these patients may have concerns regarding whether the donation will result in a live birth. Current psychological pre-transplantation assessment of LDs has primarily focused on determining suitability (Järvholm *et al.*, 2015a). As observed in the original Swedish trial, examples of medical consequences in three of the nine LDs during the first post-donation year included ureteric-vaginal fistula, transient occurrences of nocturia and thigh-sensibility impairment (Kvarnström *et al.*, 2017).

Quantitative data, collected pre-surgery and for every 3 months for the first year post-UTx, measured psychological well-being (PGWB), relationship distress, health-related quality of life (HRQoL) and mood (Kvarnström *et al.*, 2017). The baseline scores exceeded the normative values for the Swedish population. Scores for PGWB, relationship distress, and mood did not show any negative deviations during the post-hysterectomy year. A follow-up study at years 2 and 3, showed that uterus donation does not, in general, negatively affect HRQoL, mood, or relationship satisfaction (Järvholm *et al.*, 2019). However, slight negative deviations in HRQoL were found in three LDs, with yet unsuccessful pregnancy outcomes of their recipients. Moreover, one donor presented with light mood depression at Year 3, and two LDs experienced slight relationship distress. The LDs were also followed 4 years after donation for medical and psychological health (Brännström *et al.*, 2022a). All were free from vaginal, urological, or gastrointestinal symptoms although two had mild symptoms of leg/buttock pain, which did not affect walking. All scores for HRQoL, mood, and marital satisfaction were comparable to those before hysterectomy. Collectively, the 4-year follow-up clearly shows no major negative effects on health secondary to uterus donation although donor psychological well-being may decrease if her donation does not lead to live birth.

### Recipient health outcomes

Graft recipients should be followed not only during graft retention but for several years thereafter, thus enabling identification of any long-term side effects of surgery, IS, pregnancy attempts, and motherhood.

The nine recipients of the Swedish trial were followed every 3 months during the initial post-transplantation year (Järvholm *et al.*, 2015b), and then annually during Year 2, 3 (Järvholm *et al.*, 2020a), and 4 (Brännström *et al.*, 2022a). At baseline, all scored equal to or better than first-time IVF patients regarding PGWB, HRQoL, mood, and relationship satisfaction (Järvholm *et al.*, 2015b), suggesting self-selection of a mentally strong cohort. During the first post-transplantation year, stable levels were seen in women with ongoing grafts although the two recipients with early graft failures scored lower in the physical component of HRQoL at 3 months, but this normalized (Järvholm *et al.*, 2015b). During Year 2 and 3, the psychological health of those with no live birth showed negative deviations in the mental component of HRQoL and anxiety (Järvholm *et al.*, 2020a). Most recipients stated continued high relationship satisfaction. The study showed that psychological strains may occur during pregnancy attempts, possibly related to failure to achieve parenthood; counseling should therefore be offered.

At Year 4 after UTx all nine initial recipients, including the two with early graft failures, were in good medical health (Brännström *et al.*, 2022a). One patient underwent corrective surgery for incisional hernia after hysterectomy and two scored low on the mental component at Year 4, possibly because one had just become a mother and the other was childless (Brännström *et al.*, 2022a). Scores for all recipients showed a decline from baseline for marital relationships but all individual scores were above the threshold indicating significant relationship distress. It was concluded that recipients show reassuring psychological stability 4 years after UTx.

Two qualitative studies, involving annual structured interviews during the 5-year period after UTx, have been performed on the recipients of the Swedish laparotomy LD UTx study. One study, focusing on self-image after UTx revealed, ’joys and frustrations of becoming a complete women’ as a master theme (Järvholm *et al.*, 2020b), with underlying subthemes of ‘a changed self-perception’, ‘a changed body’, and ‘a changed sexuality’. It was concluded that the self-image was in general positively affected. The other study explored experiences of attempting pregnancy and of motherhood, identifying an overarching theme of ‘experiencing the previously unimaginable’, with underlying subthemes of ‘the yoke of childnessness’, ‘going through the impossible’, and ‘motherhood as surreal and normal’ (Järvholm *et al.*, 2022). It was summarized that women with UTx experience the common worry about implantation failure at ET, specific worries of graft failure and, when they become mothers, they feel like other mothers, with the associated stresses and rewards.

### Recipient’s partner health outcomes

Although recipient partners are not subjected to any medical intervention, their psychological health may be affected by UTx, IVF, pregnancy attempts, as well as by parenthood or not. At baseline, the partners scored better than norm-groups regarding mood, HRQoL, and relationship (Järvholm *et al.*, 2015b). They were relatively stable in these domains during the first post-UTx year and were not negatively affected by graft failure. At the 3-year follow-up, partners had negative deviations in HRQoL when birth had not yet been achieved, despite having partners with surgically successful grafts (Järvholm *et al.*, 2020a). Most partners stated continued high satisfaction with marital relationships.

### Child health outcomes

Children born after UTx have had *in utero* exposure to IS and current data indicate that premature delivery is common (Brännström *et al.*, 2022a; York *et al.*, 2023). Understanding whether these factors affect health of the neonate, child, and adult of UTx is paramount. Two publications regarding 2-year follow-up of a total of 22 children after UTx have reported normal growth trajectories for both weight and length of infants (Schulz *et al.*, 2022; Brännström *et al.*, 2022a).

## Exit-causes in uterus transplantation

Several reasons exist as to why a transplanted uterus should be removed before the woman has achieved the desired number of children or has had no child (Table 5). The life and health of the recipient should always be the priority, even when threatened during a pregnancy before expected neonate viability. In the future, it is likely that the UTx community will face situations where the recipient refuses to undergo hysterectomy, in which case the medical team must thoroughly counsel the patient concerning the consequences, and provide continued care for IS, rejection diagnosis, and psychological support.

**Table 5.** Exit-causes in uterus transplantation.

**Graft-related**	**Cause**

	Ischemia-related graft dysfunction
	Untreatable intra-uterine infection
	Endometrial atrophy
	Irreversible rejection

**Recipient-related**	**Cause**

	Severe nephrotoxicity secondary to calcineurin inhibitor treatment
	Post-transplantation lymphoproliferative disease
	Malignancy
	Serious systemic infection with need for omission of immunosuppression

**Pregnancy-related**	**Cause**

	Malignant gestational trophoblastic disease
	Massively repeated implantation failure/miscarriages without childbirth
	Life-threatening obstetric bleeding, untreatable by conventional techniques

**Psychology**	**Cause**

	Serious psychiatric disorder
	Recipient’s wish

Graft-related causes, due to either clear signs that the uterus will never support a pregnancy or because of necrosis, are the most obvious reason for UTx-exit. The recipient-related exit-causes relate to her continued health. In cases of nephrotoxicity, it should be kept in mind that many women with MRKHs have a single kidney (Herlin *et al.*, 2020) and uterus recipients may develop donor-specific antibodies, which may cause problems if they would need future kidney transplantation.

Excessive repeated implantation failure/miscarriages may be a more challenging reason for graft removal. In the Swedish study, one patient had 16 ETs with six miscarriages and after more than 5 years post-UTx, she wished for graft removal (Karlsson *et al.*, 2022). It is known from a general IVF population that the cumulative live birth rate continues to increase for at least five stimulated IVF cycles with numerous associated ETs (Malizia *et al.*, 2009). The recommendation of uterine removal for this reason must be balanced between likelihood of live birth and duration of IS, kidney function and psychology. Indeed, an even more difficult situation for UTx-exit recommendation concerns serious psychiatric disease of the recipient, which may be a psychotic condition with delusion, resulting in inability to make a sound decision regarding graft removal.

It should be noted that more UTx-exit causes probably will enter the scene, when we gain more and more data from ongoing clinical trials and future registry reports.

## Financial considerations of uterus transplantation

### Is uterus transplantation sufficiently cost-effective?

Apart from clinical feasibility of UTx, additional questions are whether UTx is, first, cost-effective, and, second, a justifiable use of limited healthcare resources. The only detailed financial analysis was based on the first clinical LD-UTx trial and included expenses encompassing IVF costs, UTx-surgeries, postoperative complications, and sick leave for both LDs and recipients, and spanned the start of preoperative investigations to completion of post-transplantation month 2 (Davidson *et al.*, 2021). The estimated mean total cost was €74 564, apportioned as sick leave (25.7%), postoperative hospitalization (17.8%), surgery (17.1%), preoperative investigations including IVF (15.7%), anesthesia (9.7%), pharmaceuticals (7.8%), postoperative tests (4.0%), and re-hospitalization (2.0%), with recipient costs being somewhat higher than LD costs (Davidson *et al.*, 2021). Excluding sick leave costs, the total would be reduced to around €55 000. Although the calculation above is specific to a Swedish setting, this would likely be comparable in many European countries. However, in the USA, the cost would likely be 2- to 3-fold higher, based on the price differential for ART between Europe and the USA (Speier, 2011).

The costs and interventions encompassing UTx have also raised concerns regarding its feasibility in certain countries. One Dutch study estimated costs for LD UTx at €93 850, including preoperative investigations, transplantation surgeries, 2-year follow-up with IS, and hysterectomy (Peters *et al.*, 2020), but excluded costs for care during pregnancy and delivery, which are covered by general medical insurance. The authors concluded that, subject to the current estimates of costs and risks of UTx, it would be unfeasible to perform the procedure at a tertiary center in the Netherlands (Peters *et al.*, 2020). They further noted that UTx is unlikely to be covered by Dutch medical insurance given its ‘non-life-saving’ nature and attendant significant risks to both LD and recipient. As such, they concluded that the benefits of UTx may not, at present, justify the associated risks and costs. However, since costs to set up a UTx program are great, they suggested international collaborations with referral of suitable patients to established clinics with experience in UTx.

### Should uterus transplantation be publicly funded?

Regardless of whether UTx in its current state is adequately safe and cost-effective to recommend widespread clinical adoption, a separate issue concerns how to address the seemingly inevitable possibility of it becoming commonplace. In countries with a nationalized healthcare system, a key question is whether UTx should be publicly funded? Several sides of this debate have been examined, one argument against public funding being that infertility is not a ‘proper’ disease (Wilkinson and Williams, 2016). However, many organizations, including the World Health Organization, define infertility as a disease and, as Wilkinson and Williams note (Wilkinson and Williams, 2016), this argument also presupposes that only interventions seeking to address ‘proper’ disease should be funded by the state.

A stronger argument against public funding is the contention that adequate alternatives to UTx exist. Although adoption does not confer the benefits of gestating one’s genetic offspring, it does offer parenthood and is doubtlessly more cost-effective than UTx from a national health care system perspective. Nonetheless, insofar as adoption is an alternative for all infertile individuals, withholding public funding for UTx would imply that other infertility treatments, such as IVF, do not warrant funding. While surrogacy may in certain circumstances be deemed ‘sufficiently good’, it does not offer the benefits of gestation, and faces its own ethical issues such as concerns around exploitation (Wilkinson and Williams, 2016). Moreover, gestational surrogacy is not approved in many countries, including most European countries with national health care. Indeed, arguing against public UTx-funding raises familiar concerns about healthcare inequities that would invariably ensue where reproductive treatment is available only to those able to privately fund it.

### Should uterus transplantation be covered in the USA?

In the USA, the patchwork nature of health insurance is an extant challenge that naturally extends to decisions concerning UTx coverage (Polk *et al.*, 2022). Several features of the US healthcare system complicate possible coverage of UTx (Blake, 2018). Since the UTx process involves several steps, it is possible that insurers may cover only some components of the overall procedure. By analogy, some insurers will cover the costs of deliveries resulting from IVF, but not the IVF treatments. Additionally, UTx exists between transplantation medicine and ART. In the USA, transplantation medicine has typically enjoyed insurance coverage in both public and private health systems whereas ART has often not. Lastly, in the USA, there have been persistent inequities in how limited healthcare resources are distributed. Consequently, there remains the uncomfortable complexity that, in the context of many unmet medical needs that warrant attention, UTx is but one of many interventions that may be worthy of coverage (Blake, 2018).

## Ethics of uterus transplantation

Three features of UTx are central to understanding its ethical complexities (Williams *et al.*, 2018). First, while most organ transplants aim to prevent recipient mortality, UTx seeks to provide to individuals lacking it, the capacity to gestate and deliver their own future children. Consequently, UTx is often characterized as ‘life-giving’ or ‘life-enhancing’ (Umani Ronchi and Napoletano, 2021). Second, UTx possesses elements of both transplantation medicine and ART. Existing ethical frameworks developed specifically for transplantation medicine or ART, therefore, do not straightforwardly map onto the ethics of UTx (Horvat and Iltis, 2019). Third, the prospect of UTx underscores the potential moral or social significance not only of genetic parenthood, but also of gestational parenthood. None of the alternatives to UTx—adoption, gestational surrogacy, or childlessness—offer the benefit of gestating one’s own children. A key ethical question concerns the value that should be attached to gestational parenthood, and whether ethical principles, such as reproductive autonomy or procreative liberty, entail a ‘right’ to gestate (Alghrani, 2018).

### Existing uterus transplant ethical guidelines

The ‘Montreal Criteria for the Ethical Feasibility of Uterine Transplantation’, which constitute the most comprehensive ethical guidelines for UTx (Lefkowitz *et al.*, 2012, 2013), outline several requirements that should be met for individuals to qualify as eligible recipients or donors in UTx. The position of UTx as a blend of both transplantation medicine and ART is apparent in some criteria. For example, these criteria require that a recipient ‘does not exhibit frank unsuitability for motherhood’, drawing inspiration from ART ethical frameworks (Lefkowitz *et al.*, 2012; Horvat and Iltis, 2019). More generally, there is widespread agreement that the physical, psychological, and broader societal risks of UTx ought to be carefully identified and assessed (O’Donovan *et al.*, 2019). The ethical calculus of balancing benefits and harms requires consideration of the uterus recipient, her partner, a possible LD, and any resulting children (Taneja *et al.*, 2019; Mullock *et al.*, 2021).

### What is the value of gestation?

To what extent should gestational experience and, by extension, gestational parenthood be valued? Insofar as viewpoints on reproduction and family building are culturally embedded concepts, the answer will be complex and vary across different communities (Kisu *et al.*, 2011; Padela and Clayville, 2018; Farrell *et al.*, 2020). Of note, UTx recipients will undergo a unique gestational experience, for the procedure does not involve anastomosis of pelvic nerves. Thus, sensations of gestation will likely be distinct from those of a normal pregnancy (Arora and Blake, 2015).

An additional ethical issue concerns whether, by way of promoting gestational parenthood, UTx might ingrain certain reproductive social norms that are themselves problematic (O’Donovan *et al.*, 2019). For instance, many criticisms of ART note the ‘motherhood mandate’, according to which gestating children and raising them well is viewed as a social norm or even a requirement for women (Russo, 1976). By way of enabling both genetic and gestational parenthood, UTx may be critiqued on the grounds that it seeks to develop a particular family, a ‘biological nuclear family’ (O’Donovan *et al.*, 2019). In turn, concerns have been raised that UTx may both intensify the pressure for one to adhere to prevailing social norms around motherhood and exacerbate the harms experienced by those who either cannot or choose not to procreate.

### Perspectives on uterus transplantation from the public, experts, and international community

Ethical acceptability of UTx varies across populations. For individuals with MRKHs in the USA, a strong desire exists for UTx to become both affordable and available (Fischer *et al.*, 2021). Qualitative evidence from UTx recipients suggests that the procedure is worthwhile, deemed to have a positive impact on the emotional challenges associated with AUFI, and beneficial for enhancing female identity (Wall *et al.*, 2022). In a cross-sectional study of the general US public, most respondents support allowing women to undergo UTx and find it ethically acceptable (Hariton *et al.*, 2018). Of particular interest, a Japanese study revealed that if one’s daughter suffered from AUFI, 32% of female respondents may well seek to become a donor, and 37% of male respondents may well consider asking their partners to be donors (Nakazawa *et al.*, 2019). In the UK, there appears to be widespread support of UTx amongst transplant medicine and obstetrics and gynecology providers, insofar as it is deemed medically and ethically appropriate (Saso *et al.*, 2015). In Sweden, 80% of a randomly selected population of women 30–39 years of age were supportive of UTx in ART and the support for UTx was 2-fold higher than for gestational surrogacy (Wennberg *et al.*, 2016).

However, such approving attitudes are not shared by all. For instance, in the USA, only ∼45% of surveyed reproductive endocrinologists and gynecologists felt UTx to be an ethical option for AUFI patients, citing concerns of medical complications of LDs, recipients, and resulting children (Bortoletto *et al.*, 2018). Just as the value of gestation is a culturally embedded concept, the ethical acceptability of UTx will likewise depend on religious, moral, and legal particularities specific to different countries (Olausson *et al.*, 2014). For example, Barn (2021) draws lessons from commercial surrogacy to argue that UTx is primed to be exploitative of vulnerable populations and unlikely to meet the ethical standards set by The Montreal Criteria (Lefkowitz *et al.*, 2013), given likely challenges in securing proper informed consent.

### Informed consent: live versus deceased donors

Another major theme in ethical discussions of UTx concerns the importance of obtaining adequate informed consent from potential donors and families, whether LD or DD (Dickens, 2016) For LDs, a thorough discussion of the risks, benefits, and alternatives are readily apparent prerequisites including emphasis that LDs revoke all parental rights to any resulting children gestated from the donated uterus, and that a future relationship with the child is by no means guaranteed (Bruno and Arora, 2020). Indeed, since most LDs thus far have been close relatives of the recipients, special attention must be paid to social pressures and emotional burdens inherent in asking someone to be a uterus donor (Guntram, 2021). Minimizing and ideally eliminating any possibility of coercion is a highest priority (Arora and Blake, 2015). Potential for coercion is greater with donation of uterus than of kidney because an argument could be made that, unlike with the kidney, the uterus will never be needed by the potential donor if she has completed her childbearing at the time of donation.

For individuals registered as DDs, explicit consent from family or another representative is still often legally required for uterus donation, mirroring practices for transplants such as limbs and the face (Vali *et al.*, 2022). Uterus transplantation from LDs enjoys a larger track record of clinical success compared with DD-UTx (Brännström *et al.*, 2023) but raises more ethical complexities given the risks of this complex surgery (Kirby, 2021). Consequently, some have argued that if UTx from DDs and LDs achieves a future comparable level of clinical efficacy, then LD-UTx may no longer be ethically justifiable provided the DD pool can provide a sufficient supply (Bruno and Arora, 2020). However, several studies indicate that there will be a lack of DDs suitable for uterus donation (Kristek *et al.*, 2019).

### Informed consent: recipients

Uterus transplantation in its current form poses greater risks both to the mother and fetus than those associated with routine ART. The medical risks must be clearly stated and including the possible risks of rejection. In addition, it is essential to communicate the exit plan regarding removal of this ephemeral organ after a predetermined period to avoid long-term side-effects of IS or because of rejection that cannot be managed; the latter may lead to complex emotional, ethical, and medical issues regarding termination of a highly desired pregnancy, and it is important to provide counseling for a potential scenario in which a recipient wishes to retain the organ against medical advice regarding the safety of mother and fetus. The consent should also describe the difference in pregnancy experience in a UTx recipient, including the probability that she will unlikely feel fetal movement or experience contractions and other sensations normally felt when pregnant. Finally, the consent should inform the recipient of the increased likelihood of preterm birth.

### Informed consent for research versus informed consent for clinical care

In the likely event that UTx moves from being performed under a research protocol to becoming standard clinical care, special attention must be given to adapting the elements in the research informed consent to the clinical consent form for both LDs and recipients. The risks and benefits of UTx were unknown in the original research trials and were extrapolated from those of radical hysterectomy for LDs and of kidney and liver transplantation for recipients. We now have data to rely on. Although the general risks and benefits will likely remain the same, as technologies continue to improve the risks to both the LD and the recipient—including rejection and expected pregnancy outcomes may decrease—and will need to be reflected in the clinical consent form.

### Reproductive autonomy and uterus transplantation

While ensuring that properly informed consent for UTx research or clinical care is encompassed by reproductive autonomy, the scope of reproductive autonomy is much broader. Informed consent should support reproductive autonomy by ensuring the right of persons to decline unwanted interventions. Beyond informed consent, however, reproductive autonomy also includes a right not to face obstacles to access reproductive health care such as contraception (Johnston and Zacharias, 2017). These examples underscore the undeniable importance of individuals possessing the capacity to self-determine their reproductive decisions and highlight the ‘negative rights’ entailed by reproductive autonomy. In the context of UTx, some have argued that procreative liberty, a concept closely related to reproductive autonomy, may also entail a ‘positive right to gestate’ through UTx (Alghrani, 2018). Whether a positive right to gestate via UTx exists, however, depends on the healthcare context in which it is being considered, as stated by Alghrani (2018). After all, the ability to uphold a positive right to gestate may well depend on whether the UTx procedure is funded by a healthcare system. As the clinical, legal, and ethical discussions regarding UTx continue to develop, an essential task that remains will be to further explore the extent to which reproductive autonomy supports not only the removal of barriers to accessing UTx but also even a potential affirmative right to procreate via UTx.

### Arguments against the ethical acceptability of uterus transplantation

Without doubt, UTx is an extraordinary clinical feat. However, some have cautioned against quickly dismissing alternatives, such as adoption, given that existing children should enjoy family stability, which may morally outweigh the desire for genetically and gestationally related offspring (Lotz, 2018). Indeed, some question whether UTx is morally superior to existing alternatives, such as gestational surrogacy, as arguments leveraged against the latter seem to also cut against UTx with LDs (Guntram and Williams, 2018). Others suggest that UTx may not be an appropriate use of limited healthcare resources since the treatment of life-threatening conditions ought to be prioritized over expensive and non-life-saving interventions such as UTx (Balayla and Dahdouh, 2016).

### What criteria should define access to uterus transplantation?

Ethical disagreements exist around the inclusion criteria for potential donors and recipients relating to age, length of waiting time, relationship status, and prior children (da Graca *et al.*, 2021). As UTx becomes a more routine procedure, demand for available uteri will likely outstrip supply (Romanis and Parsons, 2022). Some have suggested that ‘comprehensive child-rearing capacity’ should be a component of allocation criteria (Bruno and Arora, 2020). However, any assessment of whom possesses capacity for ‘good’ parenting would likely be too arbitrary and value-laden to serve as appropriate guidance for uterine allocation (Bayefsky and Berkman, 2018). Moreover, screening potential uterus recipients for child-rearing capacity would be discriminatory, as other methods of family building do not face such equivalent scrutiny (Tonkens, 2020). A further consideration relates to whether it might be ethically permissible to incorporate the preferences of a donor in deciding who receives their uterus—that is, could there be a role for donations directed to a specific person or class of people as an allocation criterion, apart and distinct from using ‘objective’ criteria such as recipient age, health, or waiting time (Romanis and Parsons, 2022).

### Ethics of uterus transplantation in XY individuals

Heretofore, UTx has been performed in genetically XX females. In the future, UTx may be expanded to genetically XY people including transgender male-to-female people (Balayla *et al.*, 2021), to women with the complete androgen insensitivity syndrome (Sampson *et al.*, 2019), and to cis-gender males (Alghrani, 2018). Indeed, the possibility of performing UTx to assist in the gender transition process raises opportunities beyond gestation potential as, for some transwomen, the opportunity to undergo UTx could meaningfully contribute to the success of gender transition (Voultsos *et al.*, 2021). Given these possibilities, the ethical implications of broadening eligibility to allow these individuals to be UTx recipients deserve careful consideration. However, because of anatomical, hormonal, fertility, and obstetrical complexity involvements, we strongly recommend that any UTx attempt in genetically XY humans should be preceded by systematic animal research to maximize safety and efficacy, as was performed for genetically XX females (Díaz-García *et al.*, 2012).

Although the medical barriers to performing UTx in transgender women may be overcome in time, ethical, and legal clarifications are required. For example, in the UK where ET to an individual who has not been assigned female at birth is deemed illegal (LaTourelle, 1990), UTx in transgender women is out of reach (Hammond-Browning, 2019). Although the original ‘Montreal Criteria’ suggested that UTx should be limited to genetically XX individuals on the basis of inadequate research involving non-genetically XX individuals, the revised criteria clarified that there is no ethical reason to reject the possibility of UTx in a male or trans-patient (Lefkowitz *et al.*, 2013). Indeed, it should be ethically apparent that the reproductive goals of male-to-female transgender women warrant equal consideration to those assigned females at birth (Jones *et al.*, 2019). As elegantly put by Lefkowitz *et al.* (2013), the ‘principle of autonomy is not sex-specific’. Moreover, if UTx subsequently develops into a clinically accepted treatment for AUFI, any refusal to perform the procedure based solely on gender identity may well face legal, religious, and moral obstacles.

The potential expansion of UTx to transgender people also raises ethical concerns regarding the appropriate designation of parenthood (Hamidian Jahromi *et al.*, 2023). Similar issues have arisen in UK regarding transgender men who subsequently became pregnant and gave birth who, despite changing their gender to male, were assigned as legal mothers for the purpose of birth registration (Margaria, 2021). With advances in technologies, such as UTx, that have sharpened the distinctions between genetic, gestational, and social parenthood, questions remain regarding how best to map differing conceptions of parenthood onto a legal context.

## How to set up a uterus transplantation program

Guidelines specific to setting up a UTx program in the USA are forthcoming and will be in accordance with the United Network for Organ Sharing program (P. Porrett, personal communication). However, from an international perspective, we suggest the following steps, which are based on our experience and that of other collaborating centers (Table 6). Because of procedural complexities, a UTx center should be in a tertiary center, typically a university hospital, with a track record of both advanced gynecologic and transplantation surgery (Brännström, 2021). The surgical team should include gynecologic surgeons with extensive experience of extraperitoneal surgery, and transplant surgeons, preferably with expertise in pediatric transplantation. Other UTx team members should be specialists in reproductive medicine, obstetrics, neonatology, anesthesiology, nephrology, pathology, radiology, infectious diseases, and clinical psychology. An institution with a UTx program should be committed to providing long-term support and resources to the care for LDs, recipients, partners of recipients, and children.

**Table 6.** Steps for setting up a clinical uterus transplantation program.

1. Existence of tertiary center with long term experience in gynecology and transplantation surgery
2. Existence of institutional support with long term commitment
3. Theoretical studies and journal clubs
4. Surgical training in large animal model, such as sheep
5. On site observations of human UTx at experienced centers
6. Ethics approval and registration as a clinical trial
7. First UTx procedures performed on site with supervision by experienced UTx surgeons
8. Registration in international UTx registry
9. Complete clinical and laboratory data collection for research during trial
10. Analysis of data and writing of scientific papers
11. Transition into clinical procedure

UTx, uterus transplantation

Theoretical studies are important in the planning phase. These could be in the form of journal clubs involving discussion of all relevant publications on human UTx, including those on the ethics of UTx, to ensure understanding and acceptance of all ethical aspects. Surgical training in a large animal model is an important step, as stated by the Moore’s criteria of ethics for introduction of new major surgical procedures, the IDEAL concept of evaluation of surgical innovations, and in the ethical guidelines of the International Federation of Gynecology and Obstetrics on UTx (Moore, 2000; McCulloch *et al.*, 2009; Milliez, 2009). Most teams have used the sheep auto-UTx model as the preferred large animal model due to similarity with the human in terms of body size, anatomy, and caliber of uterine vessels (Andraus *et al.*, 2017; Favre-Inhofer *et al*., 2018).

In preparation for the first clinical case, the scientific trial must be registered as a clinical trial and approval of ethics committee/institutional review board must be acquired. Given the complexity of human UTx, it is recommended that the clinical transition occurs in collaboration with a UTx-team with considerable experience and repeated surgical successes. We recommend onsite observations of human UTx at the experienced center and then on-site supervision with participation by experienced UTx surgeons for the initial cases at the home center (Ayoubi *et al.*, 2022). All UTx procedures should be prospectively registered in the international ISUTx registry (Brännström *et al.*, 2023), which is the most important tool for development of UTx to increase its safety and efficacy. Specific data should also be collected by the team for use in future scientific publications, which are likely to come several years after the first UTx case of any trial (Brännström *et al.*, 2022a). The possible introduction of UTx after completion and evaluation of cases in the scientific trial will depend on the regulations and health economy system of each country.

## Developments within the field of uterus transplantation

Several aspects of UTx are under development or will soon be developed. Advances in robotics and non-invasive rejection diagnosis focus on safety and efficacy, whereas increasing the donor-pool and uterus bioengineering have the goal of increasing access to UTx.

### Robotics

Robotic-assisted laparoscopy, or robotics, encompasses magnified three-dimensional (3D) vision, articulated wristed instruments, tremor-reduction, fluorescent imaging, and excellent surgeon ergonomics. These characteristics enable precise surgical dissections in narrow spaces, such as the pelvis (Fig. 3). Robotics was introduced into UTx in 2015 by a fully robotic LD hysterectomy (Wei *et al.*, 2017) with extraction through the vagina (L. Wei, personal communication). The surgical duration was only 6 h, possibly because the utero-ovarian veins, and not the deep uterine veins, were used as venous outflows. The Swedish team then performed a stepwise development of surgery for robotics-assisted laparoscopy in donor hysterectomy, involving eight cases from 2017, and with conversion to laparotomy for the last parts of retrieval surgery and laparotomy surgery in the recipient (Brännström *et al.*, 2022a). A similar procedure was performed in France (Ayoubi *et al.*, 2022). Since then, fully robotic LD hysterectomy has been used in five procedures in the USA (Johannesson *et al.*, 2021a) and single cases in Spain (Carmona *et al.*, 2021) and Brazil (Vieira *et al.*, 2021). The advantages in outcomes versus laparotomy were decreased blood loss, shorter hospital stay and shorter time to resumption of normal activities (Dahm-Kahler *et al.*, 2021). However, surgical durations in these cases were long (around 10 h).

There have also been UTx cases by robotic surgery in the recipient. To our knowledge, the first such procedure, performed in Sweden in 2021, involved a total robotic UTx with vascular and vaginal anastomoses. The recovery was uneventful, and the patient delivered a healthy boy at planned elective Caesarean section at gestational week 37 in late May 2023 (N. Kvarnström, personal communication). However, of note, the second recent full robotic attempt by the Swedish team was unsuccessful, requiring graft removal 2 weeks post-UTx. Nevertheless, we predict that robotics will be increasingly used in LD hysterectomy, but only if the surgical duration is considerably reduced, and in recipient surgery at a small number of centers where transplant surgeons have already acquired the skills of robotic kidney transplantation.

### Non-invasive rejection diagnosis

Approximately 30% of recipients will experience rejections during Months 1–5 and approximately 20% will do so during Months 6–10 (Brännström *et al.*, 2023). Rejections are generally asymptomatic and are currently diagnosed on histopathology of cervical biopsies (Mölne *et al.*, 2017). In solid organ transplantation, several non-invasive rejection biomarkers have been identified in body fluids. The general rejection biomarkers have been lymphocyte markers, cytokines, and chemokines, with specific biomarkers for renal rejection being perforin, and granzyme B (Lo *et al.*, 2014). Studies are ongoing with multi-omics analysis of vaginal/cervical fluids to find non-invasive UTx rejection biomarkers.

### Increase of donor pool

A major limitation for translation of UTx into clinical practice concerns donor availability. A potential recipient may not have a suitable LD, who is often a close relative or long-term friend (Carbonnel *et al.*, 2020). Moreover, very few female DDs have uteri suitable for donation (Kristek *et al.*, 2019). Since the uterus has no functional role other than bearing a pregnancy, uterus donation could occur after completed childbearing. Altruistic, non-directed LD uterus donation has been practiced within trials in the USA and the Czech Republic (Fronek *et al.*, 2021; Johannesson *et al.*, 2021b). This practice could increase, especially with use of robotics for LD hysterectomy, leading to a short recovery period. However, in LDs younger than 40 years, extensive psychological evaluation regarding irreversible loss of infertility is mandatory to be certain that donors would not later regret their permanent loss of childbearing capacity. It is debatable whether women several years before menopause should be included, as was done in the USA with 20 pre-menopausal altruistic donors having a mean (SD) age of 37.7 (6.5) years (Johannesson *et al.*, 2022).

Uterus donation has also been suggested at female-to-male transgender hysterectomy (Api *et al.*, 2017). Another possibility would be to reuse a transplanted uterus after planned hysterectomy in a first recipient after a live birth. This would avoid complex LD surgery and the uterus could easily be procured with long vascular pedicles. However, chronic rejection, affecting uterine arteries, may be present (Broecker *et al.*, 2021) and the risk for conventional rejection episodes may be increased, as seen after kidney re-transplantation (Yeo *et al.*, 2017).

Another potential way to increase the donor pool is to accept donors who are beyond the general maximum donor age of 55 years. However, multimodal imaging of uterine arteries would be required in such donors (Leonhardt *et al.*, 2022) to ensure that uterine arteries have sufficient calibers. In the Swedish robotic trial, one donating mother was 62 years at donation and the donation resulted in live birth at uterine age of 64 years (Brännström *et al.*, 2020b).

### Bioengineered uterus

The objective of using a bioengineered uterus is to bypass the hurdles of organ shortage and the need for IS by using a scaffold, which is colonized by the patient’s own stem cells to generate patient-specific uterine material. During the last decade, research on uterus bioengineering has been conducted in rat, rabbit, pig, and sheep (Frances-Herrero *et al.*, 2022). Both synthetic and biological decellularized scaffolds have been tested. In a decellularization process, with the possibility to preserve the vascular conduit network, cells and DNA, but not the extracellular matrix, are removed by detergents (Hellström *et al.*, 2014; Tiemann *et al.*, 2020). Recellularization in a bioreactor typically takes several weeks and enzymatic preconditioning can increase efficiency (Padma *et al.*, 2021). So far bioengineered uterine segments, but not complete uteri, have allowed pregnancy in the rat (Hellström *et al.*, 2016) and rabbit (Magalhaes *et al.*, 2020). We predict that it may take at least a decade before transplantation of a human bioengineered uterus can be tested.

## Conclusion

Before the first birth after UTx was achieved in 2014 (Brännström *et al.*, 2015), AUFI was regarded as untreatable. Through a translational approach during the last two decades, all facets of UTx have developed, from animal studies in rodents through domestic species and NHPs, to culminate in the completion of clinical trials. This field, underscored by extensive pre-clinical and clinical research, is an example of a methodical and scientifically controlled process for introduction of a major surgical innovation to the human, which follows the IDEAL concept for such introductions (McCulloch *et al.*, 2009). As experience increases, safety and efficacy for the LD, recipient and child will continue to improve, and costs will likely decrease. Although we are garnering details on risk and benefits of the procedure, UTx is still a clinical intervention that is debated among medical ethicists. While such debate will probably continue as new patient groups are deemed eligible for the procedure, the accumulated findings and analyses of ongoing trials and scientific developments within the field may pave the way for UTx to become an accepted infertility treatment for AUFI.

## Data Availability

There are no new data associated with this article.
